# Margetuximab: An active alternative for later‐line therapy in patients with HER2‐positive advanced breast cancer

**DOI:** 10.1002/mco2.322

**Published:** 2023-07-04

**Authors:** Yunxiang Zhou, Xiaojia Wang, Yiding Chen

**Affiliations:** ^1^ Department of Breast Surgery and Oncology The Second Affiliated Hospital, Zhejiang University School of Medicine Hangzhou Zhejiang China; ^2^ Cancer Institute (Key Laboratory of Cancer Prevention and Intervention, China National Ministry of Education) The Second Affiliated Hospital, Zhejiang University School of Medicine Hangzhou Zhejiang China; ^3^ Department of Breast Medical Oncology The Cancer Hospital of the University of Chinese Academy of Sciences (Zhejiang Cancer Hospital), Institute of Basic Medicine and Cancer (IBMC), Chinese Academy of Sciences Hangzhou Zhejiang China

1

Recently, the final results of the SOPHIA phase 3 trial disseminated in *Journal of Clinical Oncology* by Rugo et al. demonstrated no overall survival (OS) benefit of margetuximab plus chemotherapy over trastuzumab plus chemotherapy in patients with pretreated human epidermal growth factor receptor 2 (HER2)‐positive advanced breast cancer (ABC),^1^ despite the statistically significant improvement in progression‐free survival (PFS) previously reported in *JAMA Oncology*.^2^

Margetuximab is a second‐generation anti‐HER2 immunoglobulin G1 (IgG1) monoclonal antibody. Margetuximab binds the same epitope as trastuzumab, with similar Fc‐independent antiproliferative effects, yet it is Fc‐engineered for enhanced binding affinity with activating Fcγ receptor (FcγR) CD16A (FcγRIIIA) and reduced binding affinity with inhibitory FcγR CD32B (FcγRIIB).^2^ HER2‐targeted antibodies combat HER2‐positive tumor cells through multiple mechanisms, including the activation of antibody‐dependent cell‐mediated cytotoxicity (ADCC), which occupies an important position. The ADCC pathway depends on the mutual effect of the Fc domain of antibodies with FcγRs expressed by immune effector cells such as natural killer cells (Figure 1B–D).^3^ Correspondingly, the affinity of anti‐HER2 antibodies to FcγRs can predict individual clinical benefit. For instance, the presence of valine (V) instead of phenylalanine (F) at amino acid 158 of CD16A enhances the affinity for IgG1, and many lines of evidence have demonstrated that trastuzumab favors patients with the high‐affinity homozygous V allele over those with lower affinity FV and FF alleles.^3^ Remarkably, CD16A‐158F allele carriers represent 80%–90% of the global population.^4^ This underlies the Fc engineering of margetuximab to improve the binding of all CD16A V/F alleles. As enhanced trastuzumab activity was observed in CD32B‐deficient mice, the Fc region of margetuximab was also modified for decreased CD32B binding.^4^ Overall, the optimized Fc domain enhances margetuximab‐induced ADCC in HER2‐positive tumors even with low HER2 expression, thereby improving the curative effect regardless of the FcγR variant.

**FIGURE 1:**
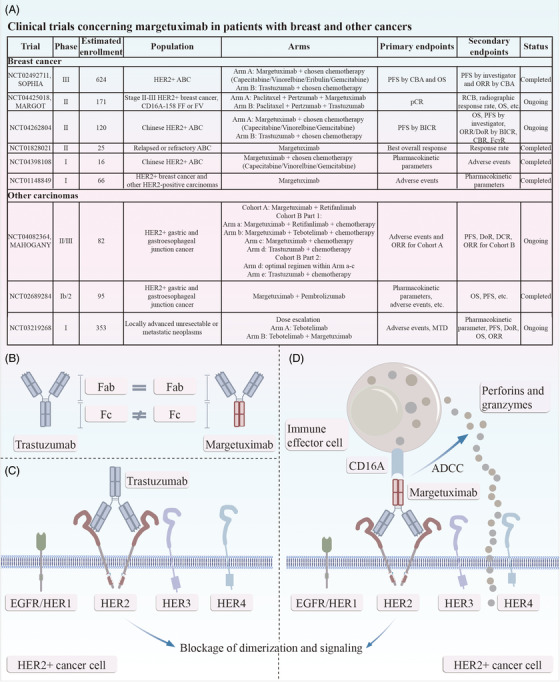
(A) Clinical trials concerning margetuximab in patients with breast and other cancer. (B) Margetuximab shares epitope specificity with trastuzumab, but with a modification of the Fc domain: five amino acids are altered from wild‐type immunoglobulin G1 to improve binding affinity for CD16A (FcγRIIIA), a stimulatory FcγR, and to reduce binding affinity for CD32B (FcγRIIB), an inhibitory FcγR. (C) Trastuzumab inhibits dimerization of HER2, thus downregulating downstream signaling, which induces the proliferation, cell‐cycle progression, survival, and invasiveness of cancer cells. (D) The Fc engineering of margetuximab leads to an enhanced ADCC activation against HER2‐positive cancer cells. ABC, advanced breast cancer; ADCC, antibody‐dependent cell‐mediated cytotoxicity; BICR, blinded independent central review; CBA, central blinded analysis; CBR, clinical beneficial rate; DCR, disease control rate; DoR, duration of response; EGFR, epidermal growth factor receptor; FcγR, Fcγ receptor; HER, human epidermal growth factor receptor; MTD, maximum tolerated dose; OS, overall survival; ORR, objective response rate; pCR, pathological complete response; PFS, progression‐free survival; RCB, residual tumor burden.

Many clinical trials of margetuximab have been carried out (Figure 1A). Rugo and colleagues conducted the SOPHIA trial, a multinational, open‐label, randomized phase 3 study to compare the clinical effectiveness of margetuximab versus trastuzumab in patients with HER2‐positive ABC after progression on two or more lines of HER2‐directed therapies and at least one non‐hormonal therapy for metastatic cancer. A total of 536 patients participated, all of whom had previously received trastuzumab. All but one had received pertuzumab, and 489 (91.2%) patients had been treated with ado‐trastuzumab emtansine (T‐DM1). The patients enrolled were randomly assigned to either margetuximab (*n* = 266) or trastuzumab (*n* = 270), both with investigator‐selected single‐agent chemotherapy (capecitabine, gemcitabine, vinorelbine, or eribulin). Primary endpoints in this trial were PFS by central blinded analysis (CBA) and OS, while secondary endpoints included investigator‐assessed PFS and CBA‐assessed objective response rate (ORR). As a final result, margetuximab plus chemotherapy prolonged PFS over trastuzumab plus chemotherapy (5.7 vs. 4.4 months by investigator, *p* = 0.001), with a 27% relative risk reduction. However, no statistically significant advantage in OS was seen (median: 21.6 months in margetuximab recipients vs. 21.9 months in trastuzumab recipients, *p* = 0.620). In addition to PFS, the ORR in the margetuximab group was higher than that in the trastuzumab arm (26% vs. 14% by investigator, *p*‐value was not provided). The PFS and ORR findings were similar to those in the previous analysis.^2^ The safety profile of margetuximab plus chemotherapy was comparable to that of trastuzumab plus chemotherapy, which is acceptable in practice.^1^, ^2^

Rugo et al. also conducted an exploratory evaluation of the effect of FcγR variants on efficacy. At the time of the final analysis, median OS was nominally prolonged by 4.4 months with margetuximab versus trastuzumab in CD16A‐158FF patients (*n* = 192, 38%). In contrast, the median OS was nominally shortened by 9.1 months with margetuximab among CD16A‐158VV homozygotes (*n* = 69, 14%). In summary, altered CD16A genotyping might drive a shift in OS advantage between patients receiving trastuzumab and margetuximab.^1^ The previous subgroup analysis nevertheless suggested a statistically significant improvement in PFS in favor of margetuximab among CD16A‐158F carriers (median: 6.9 vs. 5.1 months, *p* = 0.005).^2^

No correlation between survival benefit and CD32A/CD32B genotype was observed in the SOPHIA study. Moreover, margetuximab did not confer PFS or OS benefit in patients with CD16A‐158VV, which is inscrutable, considering that margetuximab possesses improved affinity for both alleles of CD16A compared to trastuzumab. Of note, the unfavorable prognostic profile was imbalanced between the two treatment groups, albeit without other clear explanations.^1^, ^2^ The role of margetuximab should be further explored to select the appropriate patients. To date, the utilization of CD16A allelic variants to predict the effectiveness of margetuximab is still of great concern. A phase II bridging study of SOPHIA in Chinese patients (SOPHIA CHINA, NCT04262804) is ongoing to further evaluate the efficacy and safety of margetuximab combined with chemotherapy in patients with pretreated HER2‐positive ABC in China, in which efficacy by FcγR genotype is a secondary outcome measure. Importantly, the initial results of SOPHIA CHINA reported in the 25th Chinese Society of Clinical Oncology (CSCO) Annual Meeting roughly disclosed the consistency of safety and efficacy, showing the success of the bridging. The ongoing MARGOT phase 2 randomized open‐label trial (NCT04425018) will compare neoadjuvant margetuximab to trastuzumab (both with pertuzumab plus paclitaxel) in patients with stage II–III HER2‐positive breast cancer who are low‐affinity CD16A‐158F carriers. Further exploration is warranted to determine which FcγR to use with which anti‐HER2 regimen and in what setting.

Generally, patients with HER2‐positive ABC receive multiple lines of therapy, yet rarely achieve a cure. In the epoch‐making CLEOPATRA trial, patients with HER2‐positive ABC who had not received prior trastuzumab achieved an unprecedented improvement in OS with docetaxel plus trastuzumab and pertuzumab, which established the dual HER2 blockade by trastuzumab and pertuzumab as first‐line treatment for HER2‐positive ABC. As for second‐line regimens, T‐DM1 used to be the standard treatment internationally on the basis of the EMILIA trial, which found that T‐DM1 could obtain remarkably better PFS and OS data than lapatinib plus capecitabine. Pyrotinib is also preferentially recommended by the Chinese guidelines according to the findings of the PHENIX and PHOEBE trials. More recently, the DESTINY‐Breast03 phase III study demonstrated superior PFS with trastuzumab deruxtecan (T‐Dxd, DS‐8201) compared to T‐DM1 in the second‐line setting, leading to the update of T‐Dxd as the only second‐line recommendation in the latest ASCO guideline.^5^ However, there is no standard protocol for third‐line or greater anti‐HER2 treatment in patients with HER2‐positive ABC. The treatment options for these patients include T‐DM1, T‐Dxd (if either not previously administered), tucatinib (all three strong recommendations), and so forth, which have arisen as active regimens with varying levels of efficacy, and all with prominent toxic side effects.^5^ Nevertheless, the physical conditions of patients in this setting are always poor. Hence, for populations who are unwilling or unable to tolerate these toxic effects, margetuximab has emerged as an effective alternative without increased myelosuppression, gastrointestinal effects, or cardiotoxicity. Thus, margetuximab combined with chemotherapy was approved by the US Food and Drug Administration in December 2020 and has hitherto been recommended as a later‐line scheme by most clinical guidelines. Analogously, initumumab (Cipterbin, the Chinese first‐in‐class Fc‐modified HER2‐targeted antibody) combined with vinorelbine exhibited marked efficacy and sound safety in HER2‐positive metastatic breast cancer in the HOPES trial, and this indication has been approved in China.

Overall, although margetuximab plus chemotherapy provided no OS benefit in the SOPHIA population compared with trastuzumab plus chemotherapy, margetuximab as post‐second‐line therapy offers an active alternative for patients with HER2‐positive ABC, and the findings of the SOPHIA study open the way to new lines of research aimed at developing Fc‐engineered anti‐HER2 antibodies for HER2‐positive breast cancer and other HER2‐positive carcinomas.

## AUTHOR CONTRIBUTIONS

Y.X.Z. wrote the manuscript. X.J.W. and Y.D.C. reviewed and modified the manuscript. All authors read and approved the final version of the manuscript.

## CONFLICT OF INTEREST STATEMENT

The authors declare that they have no conflicts of interest.

## ETHICS STATEMENT

Not applicable.

## Data Availability

Not applicable.
